# Guillain-Barre Syndrome After Minimally Invasive Transforaminal Interbody Fusion: A Case Report

**DOI:** 10.7759/cureus.6222

**Published:** 2019-11-23

**Authors:** Kingsley Abode-Iyamah, Angela M Bohnen

**Affiliations:** 1 Neurosurgery, Mayo Clinic, Jacksonville, USA

**Keywords:** guillain-barre syndrome, mis tlif, gbs, ascending weakness, ascending parasthesia, ascending paralysis, transforaminal lumbar interbody fusion

## Abstract

Guillain-Barre syndrome (GBS) is an autoimmune disorder in which an individual’s immune system attacks the peripheral nerve myelin. Although rare, but serious, the syndrome typically starts with numbness, tingling, or weakness in the lower extremities and progresses in an ascending fashion. Severe weakness can transmit into paralysis and respiratory compromise. Although rare, GBS has been reported as a complication of multiple surgeries including orthopedic, cardiovascular, transplant, and general surgeries. To our knowledge, we here present the first case report of GBS after minimally invasive transforaminal interbody fusion. Furthermore, we highlight the importance of understanding the presenting symptoms and identifying proper examination findings, particularly in the setting of confounding factors, for prompt diagnosis, treatment, and reduction of morbidity.

## Introduction

Guillain-Barre syndrome (GBS) is a rare immune-mediated disorder which often occurs following an infectious disease, affecting 0.8 to 1.9 per 100,000 persons per year [1]. Although the exact etiology remains unclear, it commonly occurs following a gastrointestinal infection with Campylobacter jejuni [2,3]. In addition, GBS has been associated with other infectious diseases such as mycoplasma pneumonia, haemophilus influenza, cytomegalovirus, Epstein-Barr virus, and post-vaccination [4-8].

Post-insult, an autoimmune response is initiated; antibodies that attack myelin protein are produced, causing both axonal and nerve sheath damage [9]. Patients typically present with symptoms of polyneuropathy with ascending paresthesia, weakness, autonomic dysfunction and even respiratory failure [6,7]. Post-surgical GBS has been reported following gastrointestinal surgery, cardiac surgery, thoracic surgery, and orthopedic surgery [10]. There are few reported cases following open spinal surgery [11-16]; however, to our knowledge, there is no reported case following minimally invasive spinal transforaminal interbody fusion (MIS TLIF). Here we present a unique case of GBS following MIS TLIF.

## Case presentation

**Figure 1:**
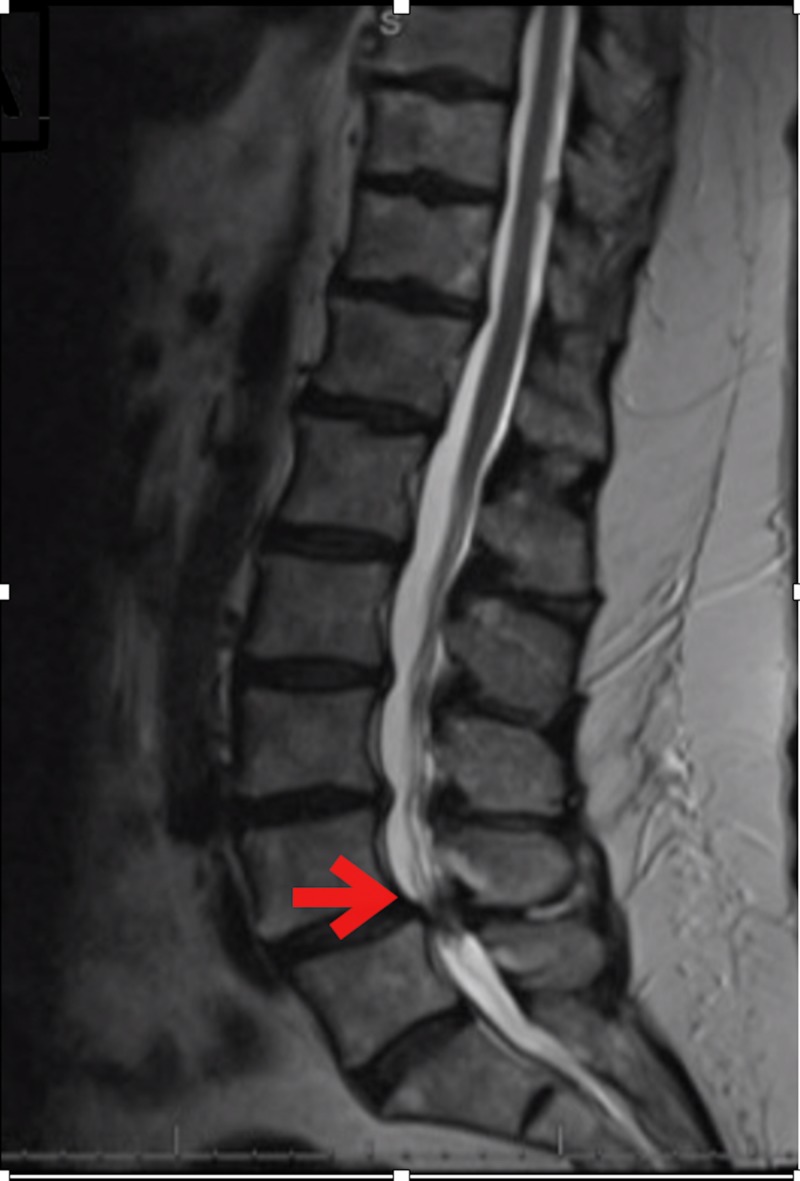
Pre-operative sagittal T2-weighted MRI demonstrating grade 1 spondylolisthesis at L4/5 with severe canal stenosis.

A 68-year-old woman with a history of breast cancer (prior lumpectomy and radiation), hypertension, and past surgical history of cholecystectomy, appendectomy, and right knee replacement presented with a history of back and leg pain. She referred to the emergency room due to an acute exacerbation of progressive back pain and neurogenic claudication. On examination, she had full muscle strength with some paresthesia in bilateral lower extremities. An MRI of the lumbar spine revealed grade 1 spondylolisthesis and severe canal stenosis at lumbar segment four/five (L4/5) (Figure 1). 

She underwent MIS TLIF at L4/5 with bilateral facetectomy and decompression without complication. Intraoperative somatosensory evoked potential/electromyography were uneventful. Immediately, post-operatively, the patient’s paresthesias and radicular pain resolved with full motor strength. On post-operative day 5, she reported subtle weakness (4+/5) which progressively worsened over the 24 hours. At this time, her examination demonstrated lower extremity areflexia, numbness, and weakness in bilateral lower extremities (graded 2-3/5). The patient also reported subjective numbness in bilateral upper extremities and episodes of dyspnea; however, her respiratory rate and upper examination were normal. She denied facial symptoms.

**Figure 2:**
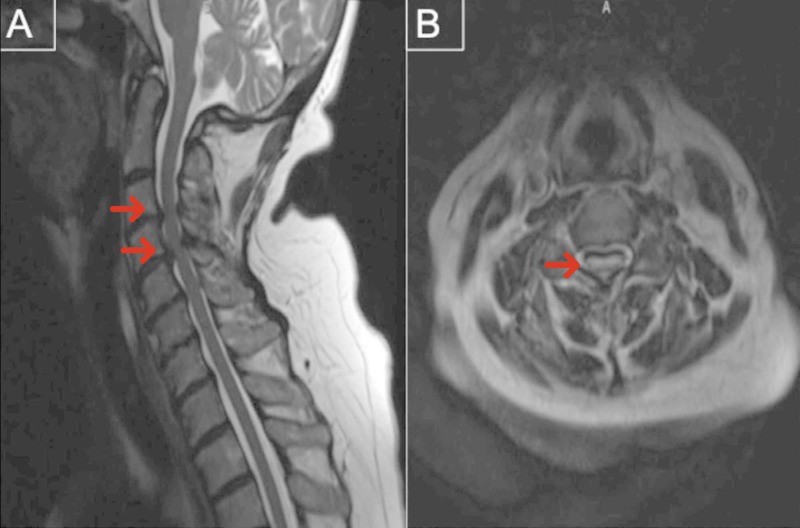
T2-weighted MRI of the sagittal (A) and axial (B) cervical spine revealing severe spinal cord compression at C4/5 with intramedullary spinal cord T2-signal.

**Figure 3:**
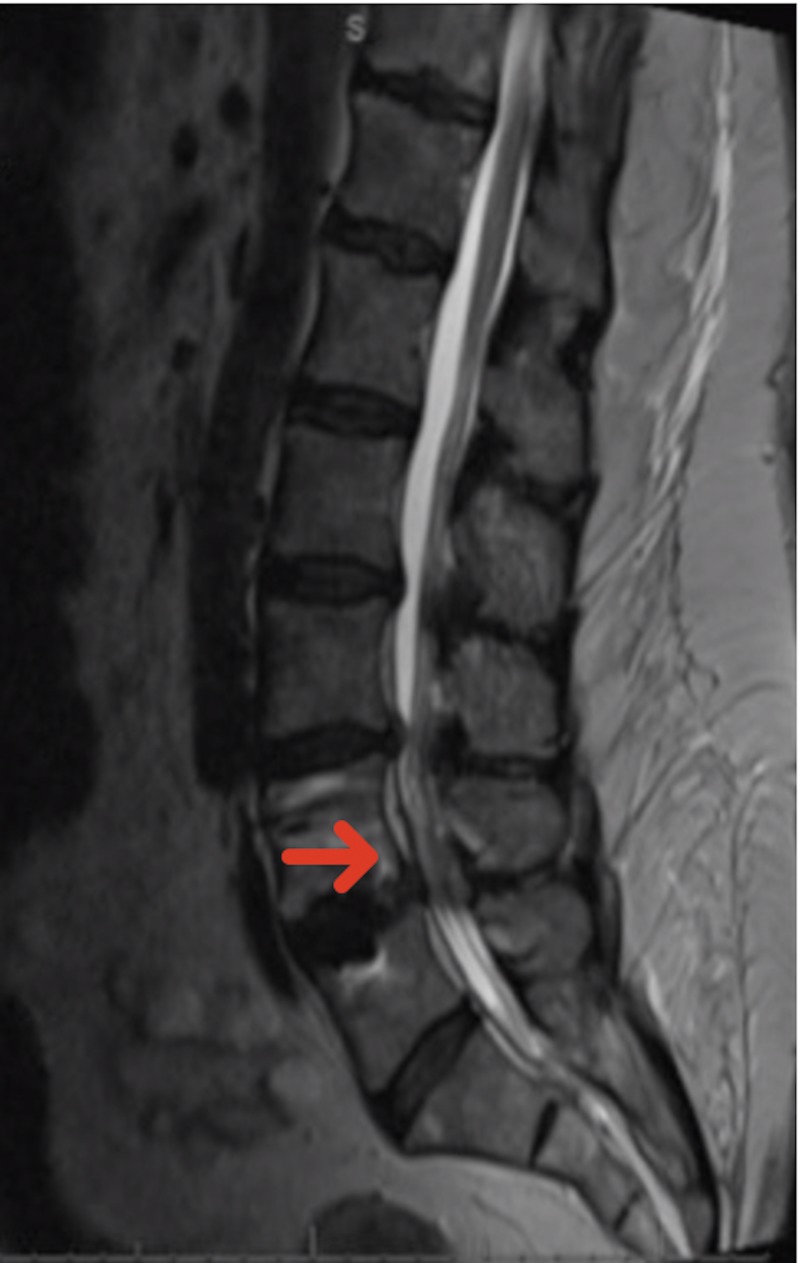
Post-operative sagittal lumbar MRI demonstrating improvement of the previous L4/5 spondylolisthesis and canal stenosis.

She underwent an MRI of the entire spine which revealed a cervical 3/4 cord compression and cord signal change (Figure 2) and post-surgical decompression at the L4/5 level with instrumentation (Figure 3). The patient underwent lumbar puncture for cerebral spinal fluid (CSF) analysis, revealing protein of 257 mg/dL (high), nucleated cell count of 1 cell/mcL, and red blood cell count of 24 cells/mcL. Given the examination and laboratory findings, she was diagnosed with GBS. 

The patient was treated with five sessions of plasmapheresis. She experienced some improvement of her symptoms during treatment and was eventually discharged to inpatient rehabilitation. At three months of follow-up, the patient demonstrated significant improvement; she was ambulatory with a walker, without objective leg weakness, and only slight paresthesia of bilateral lower extremities. Her pre-operative pain remained resolved. She continued to progress and at her six-month follow-up, she was ambulatory without aid and without paresthesia.

## Discussion

GBS is an uncommon immune-mediated polyneuropathy whose etiology is not completely understood. It is hypothesized that it results from autoimmune antibodies and inflammatory cell cross-reactivity with epitomes located on peripheral nerves, leading to demyelination and axonal damage [9]. The incidence of post-surgical GBS is reported at a rate of 5%-9.5% although it has only been previously reported in a few cases following spinal surgery [11-16].

While there are a few reports of GBS after open spinal surgery [11-16], the current case report differs from the previously reported cases in numerous ways. First, we present a case of GBS following MIS TLIF, which has never been reported. Additionally, given the confounding factor of cervical compression, our case demonstrates the importance of careful clinical examination and history-taking in order to provide an accurate diagnosis of post-operative GBS. This patient had confounding cervical compression noted on the MRI screen post-operatively (Figure 2). While there was cord signal change, her presentation and examination did not suggest an upper motor neuron lesion. This, in addition to the ascending nature of her paresthesia and weakness, led to the proper diagnosis and haste treatment. Inaccurate, or delayed diagnosis, would have increased the potential morbidity and mortality associated with the syndrome [6]. In this case, even in the setting of cervical compression we were able to make the diagnosis by a thorough physical examination and CSF analysis. 

Additionally, although recurrence of GBS only occurs in 3% of patients, re-introducing the patient to the initiating factors may further exacerbate it [6,7,17]. In fact, it has been previously postulated that surgical stress activates the neuroendocrine axis and cell-mediated immunosuppression, which may play a role in promoting infections and production of cross-reactive antibodies [18,19]. The patient had undergone numerous operations previously, without event, and no known prior surgical infections. While the recurrence is low, future treating physicians will need to pay close attention to this should she require surgical intervention again. Even after resolution of the patient’s symptoms, she continued to have no evidence of cervical myelopathy. We discussed close follow-up and monitoring for evidence of cervical myelopathy prior to further surgical intervention. 

## Conclusions

Post-surgical GBS is a rare occurrence and has never been reported following MIS TLIF. Confounding factors can exist which may complicate making the diagnosis. A thorough history, physical examination, and CSF analysis are essential for making an accurate diagnosis and providing prompt treatment. 
